# Odontological Society of Pennsylvania

**Published:** 1893-11

**Authors:** 


### ODONTOLOGICAL SOCIETY OF PENNSYLVANIA.

The first regular meeting of the winter series was held at No. 1228 Walnut Street, the President, Dr. E. T. Darby, in the chair.

The President stated that there was every reason to expect interesting meetings during the winter. Papers have been promised by men whose names and standing in the profession are sufficient to satisfy the members that they will be of the most interesting character.

The President urged every member to attend and bring others as often as possible. There was no reason, he said, why the Odontological Society of Pennsylvania should not take a stand and position equal to any in the country.

After reading the minutes and resolutions upon the death of Dr. Dixon, it was suggested that some account of the Dental Congress held in Chicago might be of interest.

The President stated that through some oversight no delegates had been appointed, and asked Dr. McQuillen to give his experience.

*Dr. McQuillen.*—The Congress was a large gathering of delegates, but the financial depression kept many at home. Many of the sections were well attended, especially the one on Operative Dentistry. The papers were interesting, but, with the exception of a paper by Dr. Emil Schreier, of Vienna, who explained his method of treating infected teeth with kalium-natrium, I did not hear anything particularly new. But there were many interesting things in the other sections. The general sessions were well attended. The clinics were unfortunate in having a crowded and stuffy room; but they were otherwise successful.

Dr. Faught requested a synopsis of the treatment advocated by Dr. Schreier.

*Dr. McQuillen.*—The idea of the treatment was to introduce the preparation, which, as I understand it, saponifies the putrescent pulp; and in many cases, if there is much moisture, it burns it out entirely, leaving a clean root, which is to be removed with alcohol or peroxide of hydrogen. I have tried it in three or four cases since my return, and in a few minutes have secured results that I have not been able to get before in one application,—that is, to find the root absolutely sweet after treatment. With other remedies it has taken more time; but with two or three applications of this material it seems to entirely change the condition of things. You have to be careful in applying it, for, if too large a quantity is used, it may result in an explosion. It is better to use the rubber dam and a small quantity of this agent. I think it is worth while for every one to try this preparation. I believe there is no secret in his preparation and that the S. S. White Dental Manufacturing Company made arrangements with Dr. Schreier to furnish it.

In many cases, after extracting the pulp, Dr. Schreier has been very successful in using it where he was not sure that every part had been removed. In working it down, it would have the same result there as in a putrescent pulp. After using it, you must proceed exactly as you would with an ordinary pulp-canal. Some seemed to think that after a time it would disintegrate if it were simply put in there and allowed to stay with the idea that the pulp had been made inert.

I used it about a week ago in a lateral incisor where I found a great deal of pus, the tooth having been dead for some time. I closed it up, and have not seen the patient since, but received a note saying she was comfortable. I have never been successful in immediate root-filling in these cases. In fact, I never attempt to fill a putrescent tooth at once. With this preparation, after thoroughly cleansing the tooth, it has been sealed up for a week, making, as I think, a good test of the preparation.

*The President.*—In lieu of a regular paper for discussion, the subject, as announced on the programme, is, "In cases of congested pulps, should arsenical applications be made without preliminary treatment?"

*Dr. Faught.*—I have no facts to offer in regard to it, and never could be persuaded to do it. The principle of my practice has been to always reduce the inflammation as far as possible first, and then make the application, as I believe it to be the better practice.

*Dr. Boice.*—I have had some experience in making applications without preliminary treatment. About ninety per cent. are successful; the other ten per cent. make you regret that you tried the other ninety. It depends largely upon the temperament of the patient and the amount of inflammation. If a patient comes with the pulp exposed, and is going away for months, I would even then put in a partial treatment by applying morphia and leaving it in for ten minutes. Some years ago I practised immediate filling quite frequently, but do not do it now.

The President asked Dr. Boice if he had ever had any experience with Baldeck's Nerve Paste in connection with congested pulps.

*Dr. Boice.*—I have not. In using arsenous acid I take it for granted that it means arsenic. Any preparation that has morphia in is hardly to the question. The finer the arsenic is ground the less liable it is to cause irritation. Some of that procured at the drug-stores is entirely unfit for use. By taking it in the fingers you can feel the grains in it. I use a porcelain slab and a steel instrument to regrind it, and do so with every application I make. The preparations upon the market are generally prepared by some secret method and are not to be relied upon. Every time I have deviated from this rule I have regretted it.

*Dr. Culver.*—I always endeavor to allay the inflammation before making the application.

The President asked Dr. Faught how he allayed the inflammation of the pulp.

*Dr. Faught.*—An idea occurred to me while the other gentlemen were speaking that is, perhaps, new to the profession. It is experimental in a measure with myself, and perhaps I should say nothing about it. My ideas have been turned in this direction for the last year and a half for many reasons. You will often find that an irritated pulp will exist in the mouth of a patient of a highly nervous organization,—a high tension pulse. I have been considering the pulse a great deal in dentistry; where you get a high tension condition the application of arsenous acid at that time only increases the difficulty, causing much trouble in allaying the inflammation with morphia, which is the remedy I apply. Even then I believe great good can be obtained if, in connection with the application of morphia, you place the patient upon the use of salicylate of soda. The use of this agent has especial reference to a high tension pulse,—to highly nervous organisms, to individuals undergoing what are characterized as sedentary occupations. If we look to the excretions of such persons we shall find that they are throwing off uric acid in varying quantities. According to the highest authorities the normal excretion is in proportion of one grain of uric acid to thirty-three grains of urea. This will perhaps vary one to eighteen, one to twenty, one to forty-two. If you place the patient upon the use of salicylate of soda it renders the condition of the urine alkaline. Uric acid is thrown off from the system when the urine is acid; therefore if you place them upon salicylate of soda you throw off that quantity held back when the urine is alkaline.

I merely make these statements as a matter of scientific inquiry. I am a strong believer that we have yet to explain some dental conditions by examination of the urine, and particularly under these irritative conditions in which the nervous system is being required to carry an added burden. In connection with the morphia application and the salicylate of soda, where the latter has been used continuously for seven days or more at a time,—not less than fifteen grains four times a day,—the excretion of uric acid in proportion to the urea never varies, but without it it may prove an added irritant to the system.

*Dr. Bonwill.*—I have but one word to say to it,—Never!

The President, asking Dr. McQuillen to take the chair, said,—

"It is undoubtedly true that it is bad treatment ordinarily to apply arsenical preparations to congested pulps. An honest confession is said to be good for the soul, and I might as well make a confession.

"The hint has been thrown out by one of the speakers that they never use any secret preparations. I must confess that I do occasionally. I experiment with it, and if it works well I continue it. When I asked if he had used Baldeck's Nerve Paste I did so for the purpose of ascertaining whether the experience of others had been the same as mine in the use of that preparation. My attention was called to it about two years ago by an English dentist. It was sent to me, and not knowing its composition I hesitated to use it. One day my bottle of nerve paste had crumbled up and did not seem in proper form to apply, so I took a little of this Baldeck's paste and put it on a pellet of cotton not larger than a homœopathic pill and applied it to the pulp. The patient was suffering from pulpitis. After sealing the cavity with rosin-wax the patient was sent away, with the understanding that I should see the case within forty-eight hours. The patient came back at the appointed time and stated that no pain had been suffered. I applied it time after time in the same way without knowing its preparation or its ingredients. I have used it now for two years successfully with one single exception. I have applied it indiscriminately to all cases of congested pulps and healthy pulps wherever obliged to devitalize.

"That is my confession. What it is I do not know. It may be arsenic, but I don't believe it could be anything worse than arsenic. If it is strychnine or cyanide of potassium it would not be worse than arsenic, and I do not know that I care what it is so long as it devitalizes the pulp and does no mischief."

*Dr. Deane.*—I do not understand Dr. Boice when he says he applies arsenic alone to the pulps, the arsenic of itself being too much of an irritant. In my practice I combine as much morphia in the cavity as I possibly can with the arsenic paste. The more there is of morphia applied to such a pulp the easier it is for the patient. I would never think of applying the pure arsenic.

*Dr. Faught.*—In a personal experience with Dr. Boice I never thought the doctor used any acid, as he only used the probe and inserted it into the cavity. I believe that Dr. Boice, in making the application, used such a microscopical quantity that it made his practice successful.

*Dr. Boice.*—The inflammation should be so under control that cold water can be injected in the tooth and the patient will not object to it, and with a probe so fine that you could put it through the cuticle without knowing it. I place it in the pulp with arsenic attached to the end. It is not the case, as Dr. Deane seemed to infer, that I apply before I get it into condition. I do not apply the arsenic until I can prick into the pulp with a probe before I use it.

*Dr. McQuillen.*—How do you get the tooth in that comfortable condition?

*Dr. Boice.*—By the use of sulphate of morphia.

*Dr. McQuillen.*—So that it is not responsive to heat or cold?

*Dr. Boice.*—Yes.

*Dr. Faught.*—I have experienced that at Dr. Boice's hands.

This closed the discussion of the subject.

INCIDENTS OF PRACTICE.

Incidents of practice being next in order, the President stated that the Chairman of the Executive Committee suggested that a meeting be held some time to recount failures as well as successes, and suggested that such might be done at the present time.

*Dr. McQuillen.*—I would follow the suggestion in describing an application made to a pulp about the 5th of July. The patient came back after a proper time. In August there was still life in the tooth-pulp. She was in the office yesterday, and the fourth application was made to it, and the pulp was still quite as sensitive as it was early in July. I applied absolutely pure arsenous acid, without any morphia or anything else. There is no pain, but it is a very sensitive pulp when touched.

Dr. Long stated that nodular pulps would sometimes behave in that way; but Dr. McQuillen, in reply, said he could find no indication of any such thing.

Dr. Darby, the President, then took the floor, and said,—

"I have brought here to-night some new preparations of plastic filling-material. This was a subject that occupied a good deal of attention and elicited considerable interest at Chicago in one of the sections. I have been using for some time preparations new to me, and have brought them here, thinking they would be of interest. I speak of Poulson's cement, and one called Plombe Granite, and a cement that Dr. Tiburtius-Hirschfeld brought to me some time ago, prepared by Carl Worff, of Berlin. She says it is most used and best liked in Germany. In my hands none of the phosphate-of-zinc formulæ have ever given the satisfaction that Poulson's cement does. Most of the German cements have phosphoric acid in a crystalline form, so that you are obliged to work it before melting it. Dr. Hirschfeld told me she had discarded all in which the phosphoric acid was in solution, preferring every time the crystals or that in crystalline form. The German cements do not harden as quickly as our American cements. In proportion as there is a small percentage of water in the phosphoric solution or crystals they harden slowly; and just in proportion as they harden slowly they become durable and lasting; and as they harden quickly, in my experience, they are ephemeral in their duration; so that I have come to the conclusion that those that require the greatest amount of work in preparing them produce the best results.

"In the mouth it takes Poulson's cement from ten to fifteen minutes to harden. It should be protected with wax, or what is still better, rosin and wax. I melt it on a spatula, and pour it on the filling after it has stood from two to five minutes. It scales after a time, but does not crumble off as wax or paraffine. I have no faith in paraffine,—it scales off as soon as it becomes wet. If you let them harden under wax and rosin for a day or so, they take a finish almost like polished ivory."

The doctor then proceeded to mix the three different preparations, and made three pellets for inspection and for the purpose of showing his mode of mixing the ingredients, using a piece of platinum, shaped into a disk about the size of a ten-cent piece, upon which, over an alcohol lamp, the substances to be melted were placed.

"The Granite cement is more granular, yet, at the same time, makes a good filling. It was sent to me by Dr. Davenport, of Paris.

"The preparations can be obtained of Dr. Poulson, of Hamburg, and cost about the same as here.

"For crown-work I use Ash & Sons' cement. It sets slowly and remains in a plastic condition for a long time."

In answer to a question as to what was a fair length of time for such fillings to last, the doctor said that he thought a filling lasting two or three years without renewing would be a good one.

Dr. Bonwill being called upon, prefaced his remarks by asking Dr. Darby how long he had used the preparation he had exhibited. Dr. Darby replied that he had used it years ago, and had laid it aside because he didn't like its action; that it was only during the last few months he had begun to use it again, and he was now more willing to take the trouble required.

Dr. Bonwill then continued, saying, "Men won't take the trouble. I cannot answer for Poulson's cement. I have been using Weston's for four or five years, but I do not like it as well as at first because I do not believe Weston manipulates it any longer; still I get good results by working it more carefully and having a certain temperature, and, everything being ready, placing it in as quickly as possible. My friend Dr. Darby will not take the trouble. By using Weston's poorer cement and being more careful in putting it in and capping it, and not only capping it, but soaking it in, he will get good results. I do not want it to stick to the surface. There is where Dr. Darby makes a mistake in placing paraffine on the surface of the filling. It should be soaked into it. I can contour cases that I never before attempted except with such material as gold, etc.

*Dr. Faught.*—I cannot allow so much to be said in favor of Weston's cement without saying that I consider it the most abominable cement on the market. I did not believe it possible for Dr. Bonwill, under the most favorable circumstances, to make it so that it will stand the tests of Poulson cement.

*Dr. Darby.*—The pellets I made during the evening will be very hard in twenty-four hours, especially the Granite and Worff's, but the Poulson cement is reasonably hard already.

*Dr. McQuillen.*—There is a cement on the market to which attention has been called in the last few months, known as Richter's Harvard cement. With the exception of Poulson's, I consider it the best I have ever handled. The phosphoric acid is in a liquid form very much like glycerin, and the powder is fine and particularly well ground.

*Dr. Boice.*—The old Weston's cement was very sandy, and in mixing it it had a dirty color, but it would wear very well. The present cement is one of the poorest we have. I have tried to use it, but was obliged to give it up. I went to the gentleman who had charge of making it and asked him to mix some, which he proceeded to do, and in two or three minutes it was very hard, but in twenty minutes it was softer. I thought the fault might have been mine, but when the person who prepared it could not make it work satisfactorily I concluded it was the fault of the materials. Poulson's cement I have tried and given up, but will try it again.

The subject was passed, and the President urged all to attend and bring others. A paper on Hypnotism was spoken of as one of the probable future subjects.

Dr. Bonwill suggested that some time the members relate their experiences in Chicago, as to what they saw new and what not new in dentistry.

Adjourned.

